# The chromatin remodeling protein BRG1 modulates BRCA1 response to UV irradiation by regulating ATR/ATM activation

**DOI:** 10.3389/fonc.2013.00007

**Published:** 2013-01-23

**Authors:** Ling Zhang, Hua Chen, Ming Gong, Feng Gong

**Affiliations:** ^1^Department of Biochemistry and Molecular Biology, University of Miami Miller School of MedicineMiami, FL, USA; ^2^Perinatal Research Laboratories, Department of Obstetrics and Gynecology, David-Geffen School of Medicine at University of California Los Angeles, Los Angeles Biomedical Research Institute at Harbor-University of California Los Angeles Medical CenterTorrance, CA, USA

**Keywords:** BRG1, SWI/SNF, BRCA1, ATR/ATM, nucleotide excision repair, UV irradiation, DNA damage response,~RPA

## Abstract

The SWI/SNF chromatin remodeling complex plays a role in the repair of UV-induced DNA damage. It was proposed that chromatin remodeling activities are utilized to increase the accessibility of nucleotide excision repair (NER) machinery and checkpoint factors to the damaged DNA. It was shown recently that BRCA1 contributes to UV damage response by promoting photoproduct excision, triggering post-UV checkpoint activation and post-replicative repair. In this study, we show that BRCA1 rapidly binds to UV damage sites when cells are undergoing DNA synthesis. In contrast, two phosphorylated forms of BRCA1 do not accumulate at sites of UV damage. Depletion of BRG1, a core subunit of the human SWI/SNF-BAF complex, impairs the recruitment of BRCA1 to the damage sites and attenuates DNA damage induced BRCA1 phosphorylation. At UV lesions-stalled replication forks, BRG1 promotes RPA phosphorylation in response to UV irradiation, since UV-induced phosphorylation of chromatin bound RPA drops significantly when BRG1 is depleted in human cells. Importantly, activation of the ATM/ATR kinases is attenuated when BRG1 is depleted. We propose that BRG1 modulates BRCA1 response to UV irradiation by regulating ATM/ATR activation.

## INTRODUCTION

Endogenous and exogenous genotoxic agents continuously damage DNA, compromise its normal function and lead to genome instability. DNA damage response (DDR) mechanisms, including several DNA repair and cell cycle control pathways, protect organisms against the adverse effects of genomic insults. UV induces the formation of primarily cyclobutane pyrimidine dimers (CPDs) and 6-4 photoproducts. These UV lesions are removed by the nucleotide excision repair (NER) pathway. During DNA replication, single-stranded DNA (ssDNA) gaps are generated by the stalling of replication forks at unrepaired damage sites (Byun et al., 2005; Cortez, 2005).

The BRCA1 protein is multifunctional and critical for the maintenance of genomic stability (Huen et al., 2010). It is important to note that BRCA1 exists in multiple complexes in the cell, which contains a wide range of DNA repair and replication proteins (Scully et al., 1997; Tibbetts et al., 2000; Wang et al., 2000, 2007; Garcia-Higuera et al., 2001; Yarden and Brody, 2001). These observations suggest a role of BRCA1 in DDR, as DNA damage induces the phosphorylation of BRCA1 and causes its recruitment into nuclear foci that contain DNA repair proteins (Huen et al., 2010).

The role of BRCA1 in response to UV exposure was partially explored in previous studies, with controversial results. BRCA1 is capable of interacting with ATR, resulting in BRCA1 phosphorylation in response to UV irradiation (Tibbetts et al., 2000). It was also reported that depletion of BRCA1 decreased RPA loading on the chromatin and RPA phosphorylation upon UV exposure (Kranz et al., 2008). Thus, it seems that BRCA1 largely contributes to the recruitment of RPA complex to damaged DNA, possibly allowing efficient ATR activation. Recently, it was demonstrated convincingly that BRCA1 is also required for post-replicative repair and the triggering of post-UV checkpoint activation (Pathania et al., 2011).

The SWI/SNF chromatin remodeling complex has been shown to modulate DNA repair *in vitro* and *in vivo* after ionizing radiation and UV irradiation. The SWI/SNF complex has been implicated to play an essential role in NER of UV damage (Gong et al., 2006, 2008; Ray et al., 2009; Zhang et al., 2009a; Zhao et al., 2009). In mammalian cells, SWI/SNF-BAF complexes (Yan et al., 2005) protect cells against UV-induced DNA damage by modulating checkpoint activation and the onset of apoptosis (Gong et al., 2008; Ray et al., 2009; Zhao et al., 2009). Depletion of BRG1 results in defective CPD repair and BRG1-deficient cells exhibit a lower chromatin relaxation as well as an impaired recruitment of downstream NER factors (Zhang et al., 2009b; Zhao et al., 2009). BRCA1 was shown to interact with the BRG1-containg mammalian SWI/SNF complex BAF (Bochar et al., 2000). We hypothesized that BRG1 may regulate UV damage repair via BRCA1. In this study, we investigated whether BRG1 regulates the recruitment of BRCA1 to sites of UV damage.

## MATERIALS AND METHODS

### CELL LINES AND CELL CULTURE

MiaPaCa-2 and HeLa cells were purchased from the American Type Culture Collection. All cells were cultured in Dulbecco’s modified Eagle’s medium (DMEM; CELLGRO, Manassas, VA, USA) supplemented with 10% fetal bovine serum (HyClone, Logan, UT, USA) and penicillin–streptomycin at 37°C with 5% CO_2_.

### shRNA TRANSFECTION

To knockdown BRG1 expression in MiaPaCa-2 and HeLa cells, MISSION shRNA Lentiviral Particles packaged with vector control (Sigma Cat#: SHC001H), a small hairpin targeting BRG1 coding sequence (Sigma Cat#: TRCN0000015549), or a small hairpin targeting ATR coding sequence (Sigma Cat#: TRCN0000015549) were used. Experiments using MISSION shRNA lentiviral particles were performed following the manufacturer’s instructions.

### MICROPORE UV IRRADIATION AND IMMUNOFLUORESCENCE STAINING

For UV irradiation, cells were washed twice in PBS and irradiated with various doses of UV. The irradiation was done with a germicidal lamp with UV-C light (254 nm) in a UV crosslinker (UVP Inc.). For micropore UV irradiation, cells were grown overnight on glass coverslips. Prior to irradiation, the media were aspirated, and the cells were washed in PBS. A 5-μm isopore polycarbonate filter (Millipore) presoaked in PBS was placed on top of the cell monolayer. The filter-covered cells were irradiated with 100 J/m^2^ of UV-C using a UV crosslinker (UVP Inc.). The filter was then removed and prewarmed medium was added to allow various times for repair. Cells on coverslips were fixed with 4% paraformaldehyde (Sigma) in 0.2% Triton X-100/PBS for 20 min on ice. Cells were washed three times in PBS, and then the DNA was denatured by incubation in 2 N HCL for 20 min at 37°C. Cells were incubated in 20% fetal bovine serum in washing buffer (0.1% Triton X-100 in PBS) for 1 h at room temperature to block non-specific binding. The primary anti-CPD antibody was a mouse monoclonal, TDM-2. Primary and secondary antibodies were prepared in 1% bovine serum albumin in washing buffer. Primary antibody was incubated overnight at 4°C and the secondary antibody was incubated for 60 min at room temperature. After each antibody step, cells were washed three times for 5 min in washing buffer. Primary antibodies used here were rabbit anti-BRCA1 (1:1,000, Millipore 07-434), rabbit anti-XPC (1:1,000, Sigma), and mouse anti-CPDs (1:500). The secondary antibodies conjugated to Alexa Fluor 488 (A21303, Invitrogen) and Alexa Fluor 568 (A11011, Invitrogen) were purchased from Molecular Probes (Invitrogen, Carlsbad, CA). BrdU mouse monoclonal antibody conjugates with Alexa Fluor 488 (Invitrogen A21303) were purchased from Invitrogen. Coverslips were mounted in ProLong Gold antifade reagent with 4,6-diamidino-2-phenylindole (DAPI; Invitrogen). Images were captured by a Zeiss LSM510/UV Confocal Microscope (with 63× oil objective) at the Analytical Imaging Core Facility of University of Miami.

### IMMUNOBLOTTING

Proteins were quantified and resolved by SDS-PAGE. Proteins were then transferred onto nitrocellulose membranes and analyzed using antibodies against the relevant proteins. The antibodies employed in this study were as follows: anti-BRCA1 (Millipore 07-434), anti-phospho-BRCA1 (Ser1423; Millipore 07-635), anti-ATR (N-19; Santa Cruz Biotechnology sc-1887), phosphor-ATR (Ser428; Cell Signaling 2853), phosphor-BRCA1 (Ser1457; Millipore 07-007), ChK1 (Cell Signaling 2345), phosphor-ChK1 (Ser345; Cell Signaling 2341), RPA32 (Bethyl lab, A300-244A), Phospho RPA32 (S33; Bethyl lab, A300-246A), ATM (Bethyl lab, A300-299A), phosphor-ATM (Ser1981; Cell Signaling 4526S).

### BrdU LABELING TO EXAMINE DNA REPLICATION

HeLa cells were trypsinized, transferred onto coverslips, and incubated for 2 h for recovery. Cells were then pulse-labeled with 25 μM BrdU for 2 h prior to UV irradiation. For micropore UV irradiation, a 5-μm isopore polycarbonate filter (Millipore) was placed on top of the cell monolayer prior UV irradiation. The cells were fixed or harvested 30–45 min after treatment as indicated. BRCA1 antibody was incubated with cells overnight at 4°C. Thereafter, the cells were extensively washed with washing buffer and incubated with AlexaFluor488-conjugated anti-BrdU mouse monoclonal antibody and anti-rabbit Alexa Fluor 568 (A11011) for 1 h. The cells were stained with DAPI and analyzed for microscopy.

### CHROMATIN ISOLATION

Chromatin fractionation was prepared by lysing cells in NETN150 (150 mM NaCl, 1 mM EDTA, 20 mM Tris–HCl, pH 8.0, 0.5% Nonidet P-40, protease inhibitors) and resuspending the pellet in MNase (micrococcal nuclease) buffer containing 3 U/μl of MNase. The extract was incubated at room temperature for 10 min with constant vortexing and then centrifuged to isolate the chromatin bound proteins in the soluble fraction.

## RESULTS

### ROBUST RECRUITMENT OF BRCA1 TO SITES OF UV DAMAGE

**FIGURE 1:**
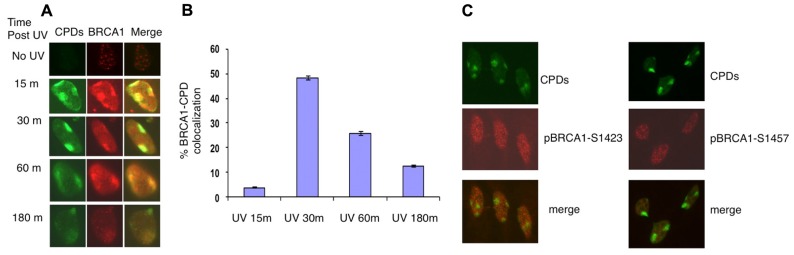
**Recruitment of BRCA1 to CPD sites**. **(A)** Asynchronously growing HeLa cells were UV irradiated (100 J/m^2^) through micropore filters. Cells were fixed at indicated time post UV irradiation and co-immunostained with antibodies recognizing CPDs and BRCA1. **(B)** Time course of BRCA1-CPD colocalization. Quantitation of BRCA1 and CPD foci in locally UV-treated cells at indicated time points. The error bars represent the SD in three separate experiments. **(C)** No accumulation of phosphorylated BRCA1 at Ser1423 and Ser1457 at sites of CPDs. HeLa cells were UV irradiated (100 J/m^2^) through micropore filters and co-stained 30 min later with CPD antibodies and antibodies specific for BRCA1 phosphorylated at Ser1423 and Ser1457 sites, respectively.

It was reported that in asynchronous, micropore UV-irradiated U2OS cells, nuclear BRCA1, and CPD staining colocalized (Pathania et al., 2011). Consistent with this finding, rapid BRCA1 recruitment to the CPD sites, as early as 15 min after UV irradiation, was detected in asynchronous HeLa cells (**Figure 1A**). We detected BRCA1 at about 45% of all CPD sites examined 30 min after micropore UV irradiation (**Figure 1B**). In contrast, only a minor portion (~5%) of CPD sites contained detectable levels of BRCA1 15 min after UV irradiation. The percentage of CPD sites with detectable accumulation of BRCA1 decreased gradually to ~25% 1 h after UV treatment. Interestingly, only ~12% CPD sites have visible BRCA1 accumulation 3 h after UV treatment (**Figure 1B**). These data suggest that BRCA1 is recruited to sites of UV damage rapidly after UV treatment. A drop of BRCA1-CPD colocalization hours after UV treatment indicates a gradual BRCA1 dissociation from sites of DNA damage.

It is well established that BRCA1 is phosphorylated in response to DNA damage. We next examined if phosphorylated forms of BRCA1 also accumulate at sites of UV damage. BRCA1 phosphorylated at Ser1423 (pBRCA1-S1423) and pBRCA1-S1457 were inspected using the micropore UV irradiation approach. Surprisingly, unlike BRCA1, neither pBRCA1-S1423 nor pBRCA1-S1457 was detectable at CPD sites (**Figure 1C**).

### BOTH BRG1 AND ATR ARE REQUIRED FOR EFFICIENT RECRUITMENT OF BRCA1 TO CPD SITES

**FIGURE 2:**
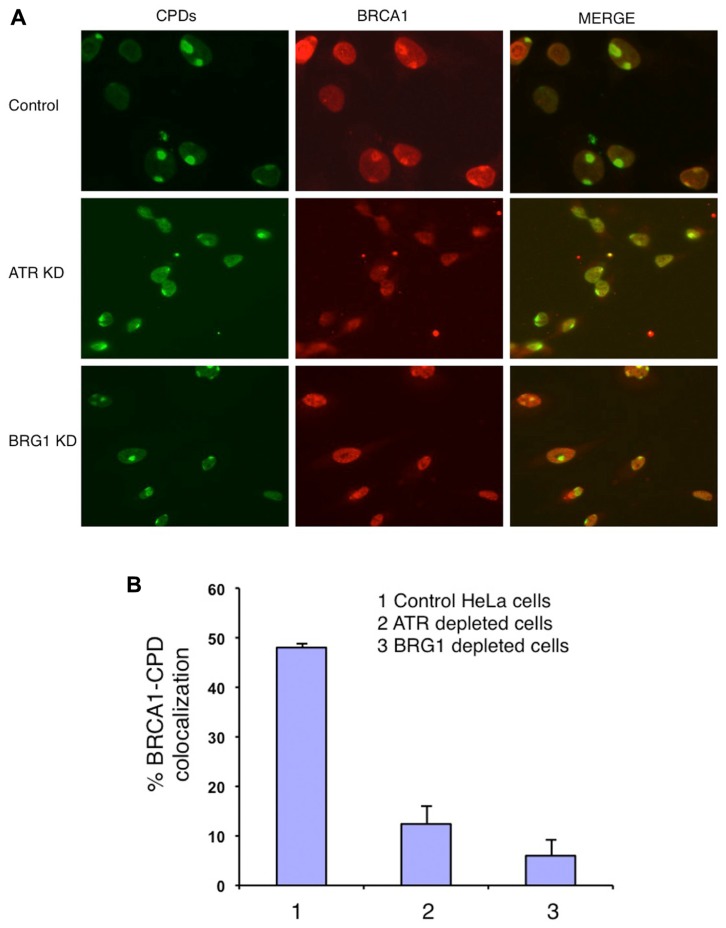
**Knockdown (KD) of BRG1 and ATR affects BRCA1 recruitment to sties of UV damage**. **(A)** BRG1-depleted cells and ATR-depleted cells showed a decrease in the number of CPD-BRCA1 foci compared to control cells 30 min post-irradiation. Experiments were performed as described in Figure 1A. **(B)** Quantitation of BRCA1 and CPD colocalization foci in BRG1-depleted cells, ATR-depleted cells compared to control cells. For each cell line, at least 150 cells with CPD foci were examined to calculate percentage of BRCA1-CPD colocalization. The error bars represent the SD in three separate experiments.

BRCA1 directly interacts with BRG1 and ATPase-deficient BRG1 (Bochar et al., 2000; Hill et al., 2004). Therefore we tested whether BRG1 depletion affects BRCA1 recruitment to the UV damage sites. We used an shRNA-based approach to deplete the BRG1 protein from HeLa cells. Cells transfected with BRG1 shRNA and control cells were then subjected to local UV irradiation treatment. In the control cells, robust accumulation of BRCA1 at CPD sites was detected 30 min after UV irradiation. In contrast, the colocalization of BRCA1 and CPDs was markedly decreased in BRG1-depleted cells, when compared to the control cells (**Figure 2A**). BRCA1-CPD colocalization foci were present in only ~8% of the CPD sties in BRG1 knockdown cells, compared to ~45% in the control cells 30 min after UV irradiation (**Figure 2B**). It is well known that ATR is activated after UV irradiation and binds to UV damage sites (Ward et al., 2004). We found that depletion of ATR has an effect similar to BRG1 knockdown on BRCA1 recruitment: BRCA1-CPD colocalization foci were present in only ~7% of the CPD sties examined in ATR knockdown cells. Our data suggests that both ATR and the chromatin remodeler, BRG1, are required for robust BRCA1 recruitment to these sites of DNA damage.

### BRCA1 RECRUITMENT IS MOSTLY DNA REPLICATION DEPENDENT

**Figure 3:**
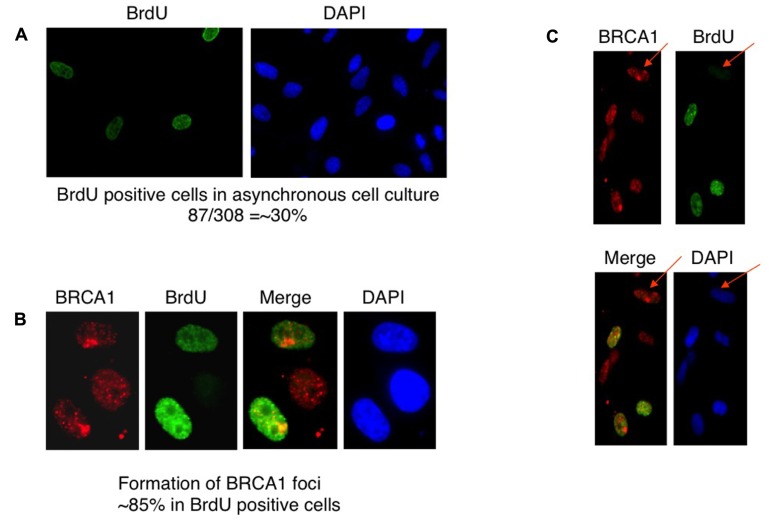
**Recruitment of BRCA1 to CPD sites is largely dependent on DNA replication**. **(A)** HeLa cells were pulse-labeled for 2 h and immunostained with anti-BrdU antibody. Asynchronously growing HeLa cells were counted to identify BrdU-positive population. About 30% of HeLa cells undergoes DNA replication, i.e., BrdU-positive. **(B)** BRCA1 foci formation after micropore UV irradiation occurs mostly in BrdU-positive cells. HeLa cells were pulsed-labeled for 2 h with 25 μM BrdUrd prior to local UV irradiation (100 J/m^2^). Cells were fixed 30 min after UV and co-immunostained with anti-BRCA1 and anti-BrdUrd antibodies. About 85% of BRCA1 foci are formed in BrdU-positive cells. **(C)** BRCA1 recruitment to sites of UV damage in non-replicating (BrdU-negative) cells. HeLa cells were pulse-labeled for 2 h and immunostained with anti-BrdU antibody. Arrows indicate BRCA1 focus formation in a BrdU-negative cell.

BRCA1 is required for the majority of photoproduct elimination in an asynchronous culture, but contributes to photoproduct elimination in a subset of replicating cells (Pathania et al., 2011). It was also shown in that study that UV damage site recruitment of BRCA1 is replication dependent. It was suggested that BRCA1 recruitment is associated with the development of stalled replication forks arising at sites containing residual photoproducts (Pathania et al., 2011). Cells were pulse-labeled with BrdU for 2 h before micropore UV irradiation. The cells were stained with anti-BRCA1 antibody and anti-BrdU antibody to determine if BRCA1 recruitment is restricted in replicating cells. Consistent with the study by Pathania et al. (2011), BRCA1 colocalization with CPD sites 30 min after UV irradiation occurred mostly in BrdU-positive cells, i.e., replicating cells (**Figures 3A–C**). We estimated that about 85% of BRCA1-CPD colocalization foci were detected in replicating cells. BRCA1-CPD colocalization does happen in BrdU-negative cells (**Figure 3C**, noted by arrow). We conclude that BRCA1 recruitment to UV damage sites is largely dependent upon ongoing DNA replication.

### BRG1 DEPLETION AFFECTS BRCA1 PHOSPHORYLATION IN RESPONSE TO UV IRRADIATION

**FIGURE 4:**
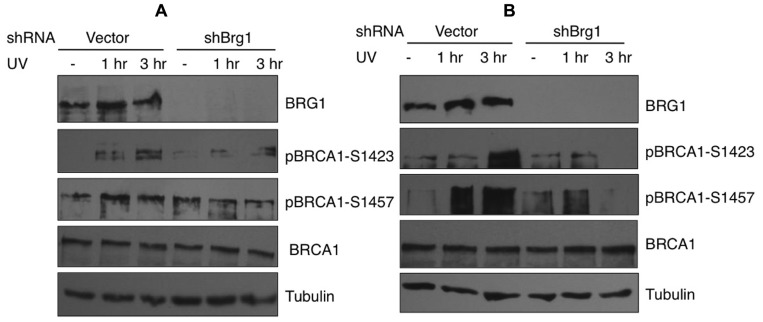
**Phosphorylation of BRCA1 at Ser1423 and Ser1457 after UV irradiation is compromised in BRG1-depleted cells**. **(A)** BRG1-depleted MiaPaCa-2 cells and control cells were exposed to 30 J/m^2^ and harvested 1 and 3 h after treatment. Cell lysates were separated on PAGE gels, immunoblotted using anti-phospho-BRCA1 (Ser1423), anti-phospho-BRCA1 (Ser1457), anti-BRCA1, anti-humanBRG1, and anti-Tubulin antibodies. **(B)** BRG1-depleted HeLa cells and control cells were exposed to 30 J/m^2^ and harvested 1 and 3 h after treatment. Cell lysates were immunoblotted using anti-phospho-BRCA1 (Ser1423), anti-phospho-BRCA1 (Ser1457), anti-BRCA1, anti-hBRG1, and anti-Tubulin antibodies.

In response to UV exposure and replication block, BRCA1 is phosphorylated by the kinase ATR on Ser1423 (Tibbetts et al., 2000). In addition it is known that BRCA1 Ser1457 is specifically phosphorylated after UV (Gatei et al., 2001). We next examined if BRCA1 phosphorylation at these sites are affected when BRG1 is depleted from cells. We employed a human pancreatic adenocarcinoma cell line, MiaPaCa-2, which contains the SWI/SNF ATPase BRG1, but lacks BRM, and HeLa cells that express both BRM and BRG1. We used RNA silencing to specifically deplete BRG1 in MiaPaCa 2 and HeLa cells. The control cells and BRG1 knockdown cells were treated with 30 J/m^2^ UV irradiation. Immunoblot analysis revealed that phosphorylation of BRCA1 at Ser1423 and Ser1457 showed significant reduction in BRG1 knockdown cells after UV irradiation, when compared to the control cells (**Figure 4**). Importantly, the cellular BRCA1 level remained unchanged after BRG1 depletion in the two cell lines (**Figures 4A,B**). Taken together, these data suggest that cells must retain BRG1 expression to ensure robust BRCA1 phosphorylation in response to UV irradiation.

### BRG1 DEPLETION AFFECTS UV IRRADIATION-INDUCED PHOSPHORYLATION OF THE RPA PROTEIN

**FIGURE 5:**
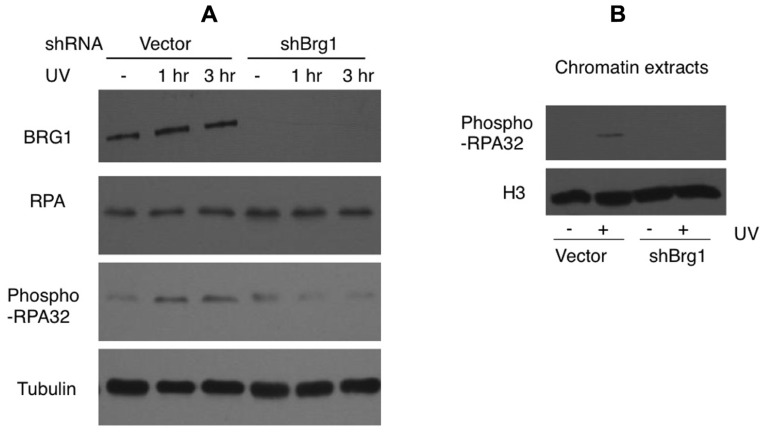
**BRG1 depletion affects UV-induced phosphorylation of RPA**. **(A)** BRG1-depleted HeLa cells and control cells were exposed to 30 J/m^2^ and harvested 1 and 3 h after treatment. Cell lysates were separated on PAGE gels, followed by immunoblotting using anti-RPA32, anti-phospho-RPA32, anti-hBRG1, and anti-Tubulin antibodies. **(B)** BRG1-depleted HeLa cells and control cells were exposed to 30 J/m^2^ and harvested 3 h after treatment. Chromatin fractions were prepared, separated on PAGE gels, followed by immunoblotting using anti-phospho-RPA32 and anti-histone H3 antibodies.

Nucleotide excision repair-independent ssDNA gaps of various lengths are generated following fork stalling at UV lesion-containing sites. They develop when the DNA polymerase at the leading strand halts at the site of damage but the helicase continues to unwind the DNA template. After UV damage, the RPA protein coats ssDNA and is phosphorylated (Carty et al., 1994). We tested if BRG1 depletion affects RPA/chromatin loading. The total cellular abundance of one RPA subunit, RPA32, did not change after BRG1 depletion, but there were significantly lower levels of phosphor-RPA32 (pSer33; **Figure 5A**). We next examined the chromatin bound levels of phosphorylated RPA32. Consistent with the overall lower levels of RPA32 phosphorylation in BRG1 depleted cells (**Figure 5A**), there was marked lower level RPA loading to chromatin (**Figure 5B**).

### THE EFFECT OF BRG1 DEPLETION ON ATR ACTIVATION

**FIGURE 6:**
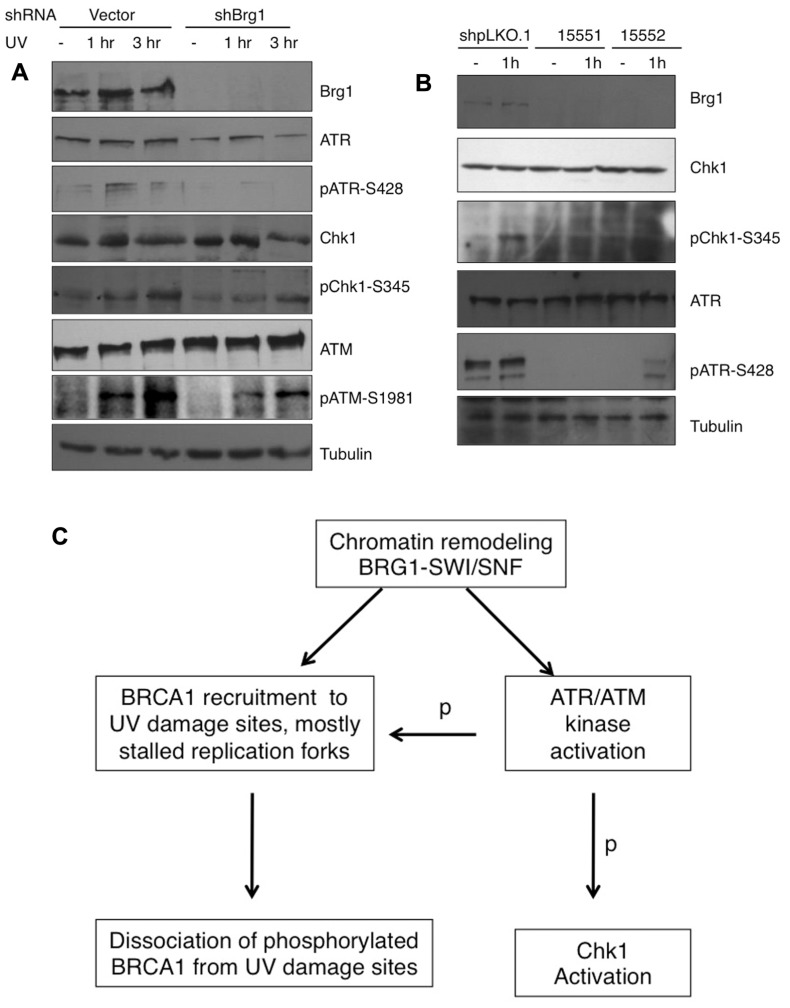
**(A)** BRG1 depletion attenuates UV-induced ATR activation and phosphorylation of ATR target Chk1. BRG1-depleted HeLa cells and control cells were exposed to 30 J/m^2^ and harvested 1 and 3 h after treatment. Cell lysates were separated on PAGE gels, followed by immunoblotting using anti-ATR, anti-phospho-ATR, anti-Chk1, anti-phospho-Chk1, and anti-Tubulin antibodies. ATM activation was also examined. **(B)** Two additional shRNAs targeting BRG1 confirms the effect of BRG1 on ATR activation. **(C)** BRG1 is an upstream regulator of BRCA1 recruitment and ATR activation. BRG1 may facilitate BRCA1 recruitment to UV-stalled forks in replicating cells, through its chromatin remodel activity. In addition, BRG1 is upstream of ATR to modulate ATR activation and phosphorylation (p) of its downstream substrates, including RPA, BRCA1, and Chk1.

RPA32 (also called RPA2) is a direct downstream target for the ATR kinase to regulate the S-phase checkpoint (Olson et al., 2006). Since BRG1 depletion has a large impact on UV-induced RPA phosphorylation, we next examined if BRG1 plays a role in the activation of the ATR pathway in response to UV irradiation/or replication stress. We found that the total cellular levels of ATR did not change significantly after BRG1 depletion in HeLa cells, but there were significantly lower levels of phosphor-ATR, a putative maker for ATR activation (**Figure 6A**). To establish that BRG1 depletion attenuates ATR activation, phosphorylation of Chk1, a direct downstream target of ATR, was investigated. Consistent with the lower levels of ATR phosphorylation in BRG1 depleted cells, Chk1 phosphorylation at Ser345 in response to UV was significantly attenuated when BRG1 was depleted from HeLa cells (**Figure 6A**). Importantly, two additional shRNA constructs targeting Brg1 transcript were used in the experiment, establishing that BRG1 depletion that leads to attenuated Chk1 phosphorylation (**Figure 6B**). These data put the chromatin remodeling protein BRG1 upstream of the ATR pathway. It is conceivable that BRG1 is required for robust activation of the ATR pathway (**Figure 6C**).

## DISCUSSION

Involvement of the SWI/SNF complex in NER prompted us to examine the role of BRG1-SWI/SNF in UV DDR. In this study, we are primarily interested in the regulation of BRCA1, an important mediator in the DDR pathway. We demonstrate that the core subunit of SWI/SNF chromatin remodeling complex, BRG1, regulates BRCA1 recruitment to DNA damage sites and RPA loading to ssDNA/chromatin. We also show that ATR/ATM activation in response to UV exposure needs the involvement of BRG1. When BRG1 is depleted from human cells, attenuated activation of ATR leads to compromised phosphorylation of RPA, Chk1, and BRCA1 (**Figure 6B**).

BRCA1 is involved in UV-induced DDR in several ways. A couple of studies suggested that UV-induced DDR is very similar to DSB-induced DDR in terms of recruitment of MDC1-53BP1-BRCA1 at the UV damage site, which promotes repair (Marteijn et al., 2009; Ray et al., 2009). It was suggested that DSBs arose through processing of the UV lesions. Recruitment and spreading of ATM along the chromatin further influence MDC1-53BP1-BRCA1 recruitment at the UV damage site, which promotes NER (Sakasai and Tibbetts, 2008; Ray et al., 2009; Yajima et al., 2009). Interestingly, a recent study showed that BRCA1’s response to UV irradiation differs fundamentally from its role in the response to DSBs. It is recruited during S/G2 to UV damage sites in a DNA replication-dependent but NER-independent manner (Pathania et al., 2011).

Consistent with these studies, we have shown here that BRCA1 is recruited to the localized UV DNA damage site rapidly and accumulation of BRCA1 at sites of UV damage peaked at 30 min after UV irradiation. This dynamic is very similar to the damage recognition protein XPC. XPC is known to play a distinct role in lesion recognition and initiation of assembly of repair machinery in GGR. We and others have reported that XPC accumulates at DNA damage sites immediately after UV irradiation and its recruitment peaks at 30 min after UV irradiation, and thereafter decreases gradually (Wang et al., 2003; Zhang et al., 2009b). In this study, using micropore UV irradiation method, we found that BRCA1 recruitment displays a pattern similar to XPC recruitment. Our view of BRCA1 function in UV DDR is, to some extent, different from Marteijn et al. (2009). These authors suggested that BRCA1 recruitment to UV damage site is a late event, represents an extra line of defense against the UV-induced damage. We believe that BRCA1 has other roles in UV damage response. We found that most BRCA1 colocalization with CPD foci occurs in replicating cells, suggesting that the protein is attracted to the stalled replication forks which contain UV-induced photoproducts. Given the replication-dependence of the process, it appears that BRCA1 is attracted to the UV-stalled forks and not simply to photoproducts.

In this study, we demonstrated that depletion of BRG1 abolished BRCA1 recruitment to the UV-induced damage sites. To support this, Ray et al. (2009) also show that depletion of another subunit of SWI/SNF, hSNF5, affects phosphor-BRCA1 foci at the UV damage sites. Recent findings suggest that both ATR and ATM directly influence BRCA1 phosphorylation at the damage site. Numerous DNA damage-induced phosphorylation sites on BRCA1 have been identified, including 1423, 1457, and 1524. Previously, pBRCA1 S1524 was reported to form clear foci at damage site even at very low dose of UV (Ray et al., 2009). Surprisingly, no foci of pBRCA1 S1423 and S1457 at UV damage sites were detected in this study, while unmodified BRCA1 forms clear foci. It is possible that, unlike pBRCA1 S1524, phosphorylated BRCA1 at S1423 and S1457 dissociates from the damage sites after being modified. It is unlikely that pBRCA1 S1423 and S1457 remained associated with the damage sites, since immunoblot results in **Figure 4** demonstrated that the levels of pBRCA1 S1423 and S1457 kept increasing 3 h after UV irradiation. In contrast, an antibody recognizing BRCA1 showed that BRCA1-CPD colocalization peaked 1 h and dropped significantly 3 h after UV irradiation (**Figures 1A,B**).

It appears that the SWI/SNF chromatin remodeling activity facilitates BRCA1 recruitment to stalled replication forks. We also uncovered the role of the SWI/SNF complex in RPA loading to ssDNA/chromatin. We observed that depletion of BRG1 leads to decreased RPA loading onto chromatin and attenuates RPA32 phosphorylation by ATR upon UV exposure. We propose that BRG1 is an upstream regulator of BRCA1 recruitment to UV damage sites. BRG1 is also upstream of ATR to modulate ATR activation and phosphorylation of its substrates, including RPA, BRCA1, and Chk1 (**Figure 6C**). BRG1 may facilitate BRCA1 recruitment to UV-stalled forks in replicating cells, through its chromatin remodeling activity. Similarly, BRG1 may help RPA loading onto ssDNA/chromatin, allowing efficient ATR activation. Taken together with the findings by Ray et al. (2009) showing the role of the SWI/SNF subunit, hSNF5, in ATM activation, we believe that BRG1-SWI/SNF modulates BRCA1 response to UV irradiation by regulating ATM/ATR activation.

## Conflict of Interest Statement

The authors declare that the research was conducted in the absence of any commercial or financial relationships that could be construed as a potential conflict of interest.
